# The Role of p53 and MDM2 in Head and Neck Cancer

**DOI:** 10.5402/2011/931813

**Published:** 2011-12-07

**Authors:** N. Denaro, C. Lo Nigro, G. Natoli, E. G. Russi, V. Adamo, M. C. Merlano

**Affiliations:** ^1^Oncology Department, ASO Santa Croce e Carle Cuneo, Via Michele Coppino 21, 12100 Cuneo, Italy; ^2^Radiotherapy Department, ASO Santa Croce e Carle Cuneo, Via Michele Coppino 21, 12100 Cuneo, Italy; ^3^Oncology Department, Policlinico G Martino, Via Consolare Valeria 1, 98100 Messina, Italy

## Abstract

Head and neck cancer is a complex disorder that includes mostly squamous cell carcinomas that can develop in the throat, larynx, nose, sinuses, and mouth. Etiopathogenesis is due to tobacco and alcohol consumption and to infection by human papillomavirus (HPV) type 16/18. Tumors often develop within preneoplastic fields of genetically altered cells. Most head and neck cancers result from multistep accumulation of genetic alterationsm resulting in clonal outgrowth of transformed cells. These DNA changes are caused by a variety of mechanisms like endogenous mutations and exogenous mutations. Dysregulated molecular pathway includes alterations of critical inhibitor of cyclin CDK complexes, inactivating mutations of p53 gene, and activation of oncogenes and growth factors. This paper attempts to review the role of p53 and MDM2 genetic aberrations and pathways in head and neck cancer.

## 1. Introduction

Head and neck squamous cell carcinoma (HNSCC) remains a major clinical challenge in oncology and represents the sixth most common neoplasm in the world today [1].

The prognosis of patients with HNSCC is not significantly improved in recent years despite the strengthening of diagnostic and therapeutic approaches. This failure is essentially due to marked clinical heterogeneity of the biological behavior of these tumors, resulting in the accumulation of multiple gene mutations, often different from each other tumor. Several unique genetic mutations combine to cause head and neck cancer. However, it is still unclear which are driver mutations, which events must occur firstly, and if a specific order is involved in molecular tumorigenesis. Additionally, the role of environmental exposure (alcohol and smoking) and viral carcinogenesis has to be clearly assessed. Recently, studies on the mechanisms underlying the deregulation of proliferation have allowed to identify several oncogenes and tumor suppressor genes involved. The tumor suppressor TP53 and its negative regulator mouse double minute 2 (MDM2) play crucial roles in carcinogenesis. P53 tumor suppressor, as a gatekeeper, plays a major role in sensing and responding to a variety of stress to maintain cellular homeostasis. Alterations in the p53 gene are described in almost all malignancies [2].

In HNSCC, p53 mutations are generally considered to be an early event in tumorigenesis which most commonly occurs in guanosine nucleotide probably due to exposure to carcinogens in tobacco smoke and also potentially as a consequence of alcohol consumption [3, 4].

## 2. Pathology

The p53-MDM2 paradigm represents the best studied relationship between a tumor suppressor gene which functions as a transcription factor and an oncogene which works as an E3 protein ligase. Lack of p53 function precludes p53-triggered apoptosis or cell-cycle arrest. Some mutations can also exert dominant negative effects on p63 and p73, two related proteins with a key role in apoptosis and differentiation [5].

The MDM2 gene is a cellular proto-oncogene amplified in 25%–40% of all human cancers. In HNSCC, the reported frequency of MDM2 expression or upregulation is high, ranging from 40% to 80% [6].

MDM2 gene maps to chromosome 12q13-14 and was originally identified as a highly amplified gene present on double minutes in a spontaneously transformed tumorigenic derivative of a Balb/c cell line called 3T3DM [7].

Human *MDM2* is 491 amino acids long and interacts through its N-terminal domain with an *α*-helix present in the transactivation domain of p53 [8, 9].

Overexpression of MDM2 can occur by increased transcription or by enhanced translation [6]. The MDM family includes MDM2 and MDMx proteins, both critical negative regulators. MDM2 is an E3 ubiquitin ligase that targets p53 for ubiquitination and degradation. In response to DNA damage, insulin, growth factors, amino acids, and energy status, MDM2 could be phosphorylated on various sites through different pathways, including p38/AKT7Mtor/S6K1 pathway and Atm/c-Abl pathway. MDM2 regulates cell proliferation, senescence, and apoptosis through targeting p53. Therefore, MDM2 is a p53 target which in turn serves to limit the amount of p53 via its efficient targeting degradation. Close spatial coexpression of p53, MDM2, and MIB1 (an E3 ubiquitin-protein ligase) immunoreactivity was observed at the invasive front of the HNSCC and in the basal and suprabasal layers of the nontumors epithelium in all p53 positive cases (Figure 2) [8].

Although p53 and MDM2 relationship is vital to regulate proliferation and apoptosis, several other proteins are implicated in the regulation of p53 stability in HNSCC, so p53-MDM2 is a central but integrated part of the complex cellular network. For example MDM2 is regulated by p14^ARF^ (on Chromosome 9p21: CDKN2A gene) which directly binds to and Inhibits the function of MDM2, thus leading to stabilization of p53. There are at least two proteins encoded from CDKN2A locus: p14^ARF^ and p16^INK4^; usually CDKN2A mutations affect p16^INK4^ or both proteins, suggesting that this is the principal susceptibility gene. In the absence of genetic damage p53 transcriptional activity is inert. About 50% of human tumors types carry a p53 mutation [2–4]. Most of mutationsare localized within the DNA-binding domain, thereby affecting p53 transcriptional activity. Inactivation of p53 function in head and neck carcinogenesis is frequently due to MDM2 binding. At the same time, MDM2 low expression is associated to mutations in p53 that prevent upregulation of MDM2 [8].

**Figure 1:**
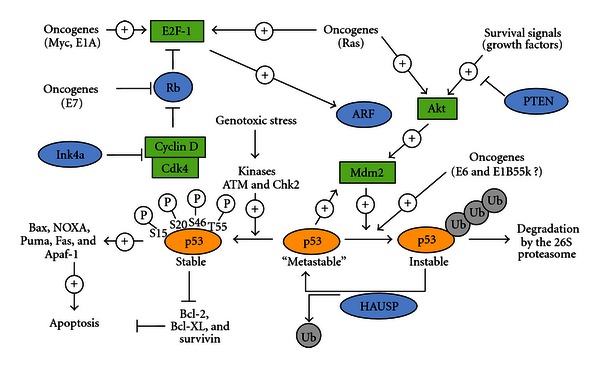
p53 and MDM2 Network: Apoptosis (http://www.celldeath.de/). In a normal growing viable cell, the p53 protein is inert. MDM2 directly interacts with p53 and thereby catalyzes ubiquitination of p53. Ubiquitination of p53 can be reversed by the action of the deubiquitinating enzyme HAUSP (also known as USP7, it is an ubiquitin-specific protease that acts as suppressor) which thereby can rescue p53 from degradation. P53 is stabilized in response to genotoxic stress such as DNA damage which leads to its phosphorylation at several specific serine and threonine residues. Phosphorylated p53 translocate into the nucleus where it activates the transcription of proapoptotic genes and suppresses the transcription of antiapoptotic genes, thus inducing apoptosis. p53-mediated apoptosis signaling is dependent on the interplay of many regulatory factors, including protooncogenes as well as tumor-suppressors. MDM2 activity is positively regulated by the action of the Akt kinase: when phosphorylated by Akt, MDM2 is able to translocate from the cytosol to the nucleus, where it unfolds its inhibitory effect on p53. Akt kinase, on the other hand, is activated in response to survival signals coming from growth factor receptors. MDM2-mediated suppression of p53 is blocked by the action of the ARF tumor suppressor. By binding to MDM2, ARF prevents the interaction between MDM2 and p53 and therefore stabilizes and activates p53. ARF expression is dependent on the transcription factor E2F-1 which is regulated by the retinoblastoma (Rb) tumor-suppressor and by the action of oncogenes. As an example, mitogenic signals lead to the activation of oncogenes such as c-myc and ras which among others activate E2F-1, resulting in increased ARF activity, stabilization of p53 and induction of apoptosis. Therefore, increased mitogenic signalling or inappropriate oncogenic activity not necessarily causes excessive proliferation but in cells with intact p53 signalling pathways can act as apoptosis inducers.

**Figure 2:**
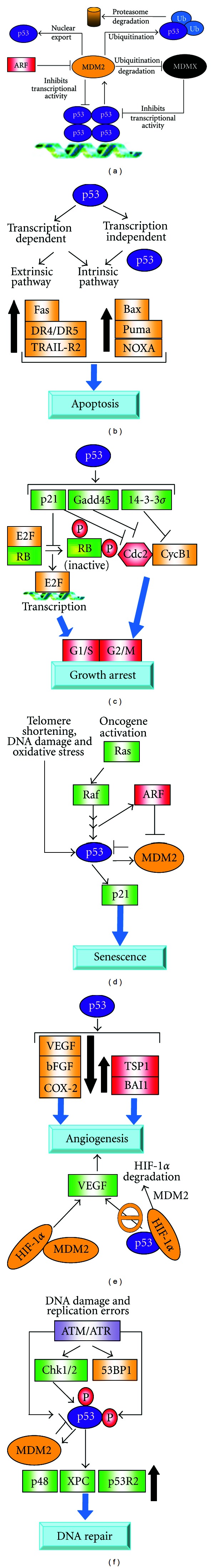
P53/MDM2 regulation [8]. (a) Regulation p53/MDM2. MDM2 inhibits p53 through an autoregulatory loop MDM2 directly binds to the transactivation domain of p53 and inhibits its transcriptional activity, inducing the ubiquitination and proteasomal degradation of p53, by exporting p53 out of the nucleus. ARF binds to MDM2 and sequesters MDM2 into the nucleolus, leading to the stabilization of p53. (b) P53 can lead to induction of apoptosis via intrinsic (mitochondrial) and extrinsic (death receptor) apoptosis pathways. (c) P53 activation can halt cell-cycle progression in G1-S and G2-M phase through p21, Gadd45, and 14-3-3-*σ* proteins. (d) P53 regulates senescence through p21-Rb-E2f signaling pathway. (e) P53 can suppress angiogenesis through the downregulation of antiangiogenenic proteins. (f) P53 plays a critical role in DNA damage repair. DNA damage and replication errors can activate ataxia telangiectasia mutated (ATM) and activate ataxia teleangiectasia and Rad kinases.

MDM2 upregulation in HNSCC has not been found to be associated with underlying MDM2 amplification (Figure 1) [9].

## 3. P53 and MDM2 as Markers of Risk, Prognosis, and Predictors of Response

P53 and MDM2 were recently studied to evaluate their role as predictors of clinical outcome. In many solid tumors, their mutations correlate with outcome. In the last decade, it has been demonstrated that the single-nucleotide polymorphism (SNP), arginine or proline at codon 72 of the p53 gene, is associated with the risk for development of various neoplasm; MDM2 SNP309, a single-nucleotide T to G polymorphism located in the MDM2 gene promoter, has been reported to correlate with cancer risk and outcome, but results, of published studies and subsequent meta-analyses, about this association remains contradictory [10–14].

It is generally accepted that HNSCC arises from a common premalignant progenitor which is transformed as a result of subsequent mutations that lead to the acquisition of a neoplastic phenotype (aggressiveness and invasion). MDM2 and p53 play an important role in carcinogenesis multistep: it has been shown that mutations in these genes are associated with dysplastic and neoplastic changes [2–4].

Girod et al. (1995) published their experience about a correlation between p53 and MDM2 mutations and grade of dysplasia. The increase in the number of p53 and MDM2 positive biopsies was correlated with loss of differentiation in the premalignant and malignant lesions. In late stages of the disease, the number of biopsies that expressed both p53 and MDM2 increased [15].

The transforming potential of MDM2 has been attributed to the overproduction of the protein. The MDM2 polymorphism SNP309 was found to increase levels of MDM2 RNA and MDM2 protein with a subsequent attenuation of p53 pathway *in vitro* [14, 16].

Increased 17p13 loss of heterozygosis (LOH) has also been documented in poorly differentiated tumors leading to the suggestion that loss of p53 function may be implicated in the transition from preinvasive to invasive HNSCC. Previous studies have demonstrated that chromosome loss at 9p21 and 10q22 and MDM2 oncogene amplification are the other more common genetic alterations in these tumors [17, 18].

Millon et al. (2001) analyzed MDM2 gene amplification, mRNA, and protein expression in tumor specimens from 62 head and neck patients. MDM2 gene amplification and mRNA overexpression was found to be infrequent, 7% and 9% respectively. MDM2 immunohistochemistry was positive in 47% of the cell, and thus, more than half of the tumors display no or low levels of MDM2 protein. In contrast, MDM2 protein was always detectable in basal and parabasal cells of morphologically normal epithelium outside the invasively growing tumor; similarly, the total amount of MDM2 transcripts analyzed by reverse transcriptase-polymerase chain reaction is reduced in tumor samples compared to normal tissues [19].

Although alterations in p53 appear to correlate to an earlier onset, probably this finding in HNSCC is primary site dependent (it seems more actual for hypopharynx and larynx rather than oral cavity oropharynx). A recent meta-analysis by Zhuo et al. reported no association between the p53 codon 72 polymorphism and risk of oral carcinoma [13].

Recently, Wan et al. (2011) performed a meta-analysis on cancer risk (27,813 cases with various tumor types and 30,295 controls) to clarify the potential interaction between MDM2 SNP309 and p53 mutational status. The data reviewed indicated that variant homozygote 309GG and heterozygote 309TG were associated with a significant increased risk of all tumor types (homozygote comparison: odds ratio (OR) = 1.25, 95% confidence interval (CI) = 1.13–1.37; heterozygote comparison: OR = 1.10, 95% CI = 1.03–1.17). Moreover, the combination of GG and TG with p53 codon 72 significantly increased the risk of cancer [20]. However, no association was reported between MDM2 SNP309 and tumor susceptibility in the stratified analysis by p53 mutational status (GG versus TT: OR = 1.17, 95% CI = 0.75–1.82 and TG versus TT: OR = 1.09, 95% CI = 0.89–1.34 for positive p53 mutational status; GG versus TT: OR = 0.95, 95% CI = 0.72–1.25 and TG versus TT: OR = 1.06, 95% CI = 0.85–1.30 for negative p53 mutational status) [21].

Yu et al. (2011) demonstrated an earlier onset of HNSCC when MDM2 promoter and p53 codon 72 are mutated. In detail, their finding suggest that both MDM2 promoter polymorphism and p53 codon 72 polymorphism may contribute to nonoropharyngeal cancer risk and that MDM2 SNP309 G-allele and p53 codon 72 SNP may accelerate the development of nonoropharyngeal cancer in women (an early age at onset of nonoropharyngeal cancer in an allele-dose response manner) [22].

In the same paper, Yu et al. published the results of six studies meta-analyses on the association between MDM2 SNP309 and the risk or age at onset in HNSCC [23–27]. They showed that MDM2 SNP309 was not significantly associated with risk of HNSCC. A possible explanation is either that the effect of the MDM2 SNP309 on HNSCC risk may be modest and could not be detected in this study, or the effect can be modified by other SNPs in other genes. Indeed, patients who carried the two to three risk genotypes (i.e., MDM2 SNP309 GT/TT, MDM2 SNP2164 AA, and p53 codon 72 CC) appeared to have an increased risk of nonoropharyngeal cancer, and this risk was more pronounced among ever smokers and ever drinkers [22].

This data has been further confirmed in the Asiatic population: in a study of 103 pts, the GG genotype of MDM2 SNP309 was associated (*P* = 0.032) with an earlier onset of HNSCC. The average age at tumor onset was 65.6 years for TT, 62.9 years for TG, and 56.7 years for GG. The patients with the GG genotype had a significantly earlier tumor onset in comparison to those with the TT genotype [24].

The prognostic role of p53 in HNSCC was firstly analyzed in a critical review by Oh and Mao (1997). They conclude that tumors overexpressing p53 tend to be more aggressive and to have the shortest survival, but most studies did not confirm such a correlation. Additionally, in patients with early laryngeal or hypopharyngeal cancers, p53 expression has no adverse effect on survival [4].

In a series of 115 patients with HNSCC immunohistochemistry (IHC) for p53, this gatekeeper loses its independent prognostic value when cyclin D1 is overexpressed. Cyclin D1 overexpression likes to be able to overcome the effects caused by p53 deletion [28]. 

Poeta and colleagues (2007) reported on a multicenter prospective analysis of p53 status and survival data for 420 cases of surgically treated HNSCC from all anatomical subsites. Mutational analysis of all of the coding exons of the p53 gene demonstrated an association between p53 mutation and survival, with p53 mutations being significantly associated with a shorter overall survival in HNSCC compared with wild-type cases. P53 phosphorylation is very important to avoid MDM2 negative regulator control; in this way, several cancers showed phosphorylation on three N-terminal (Ser15, Thr18, and Ser20) residues. In most cases, phosphorylation is associated with protein stabilization [29].

Vlatkoviä et al. (2011) analyzed the influence of p53 and MDM2 on survival. In their experience, neither p53 nor MDM2 alone were significantly associated with outcome in Kaplan-Meier analyses although MDM2 expression was found to be an independent parameter associated with increased survival (*P* < 0.03; OR = 0.63; 95% CI = 0.41–0.96) by Cox multivariate analysis. Additionally, they investigated the expression of MTBP (a-MDM2-binding protein). MTBP can contribute to p53/MDM2 homeostasis; it acts as an inhibitor of tumor progression in a subset of head and neck cancer patients. Low expression of MTBP is significantly associated with reduced overall survival in HNSCC patients [30].

Previous *in vitro* studies have shown that response of cells exposed to anticancer agents is strongly influenced by SNP at codon 72 in wild-type p53. *In vivo*, the outcome of chemoradiotherapy of squamous carcinomas is more favorable in cancers retaining a wild-type 72R allele, such cases having higher response rates and longer survival than those with wild-type 72P [20].

About clinical implications of these findings, Perrone et al. (2010) reported that the loss of function (transactivation activities) of *p53 *mutant proteins may predict a significant low pathological complete response rate and suboptimal response to cisplatin-based neoadjuvant chemotherapy in patients with oral cavity SCC [31].

The relationship between mutated p53 status and low levels of MDM2 found in cell lines is also observed to a certain extent in primary tumor samples. Overall, there is a high frequency of TP53 mutation and underexpression of MDM2 in the head and neck tumors. Moreover, a significant decreased MDM2 expression is observed in those patients with advanced tumor stage and lower 3-year survival [17, 30].

Recent studies have shown that inhibition of ribosomal biogenesis can activate p53 through ribosomal protein- (RP-) mediated suppression of MDM2 E3 ligase activity. Mutations in MDM2 that disrupt RP binding have been detected in human cancers; however, the physiological significance of the RP-MDM2 interaction is not completely understood.

Miliani de Marval and Zhang (2011) generated mice carrying a mutation that disrupts MDM2's binding to RPL11 and RPL5 to analyze this interaction. Despite being developmentally normal and maintaining an intact p53 response to DNA damage, the MDM2C305F mice demonstrated a diminished p53 response to perturbations in ribosomal biogenesis [32].

The results of some of the most impressive studies on correlation of p53-MDM2, outcome and age of onset are summarized in Table 1.

## 4. HPV and p53-MDM2 Relationship

The increasing incidence of oral squamous cell carcinoma (OSCC) correlated with human papilloma virus (HPV) led to study the influence of HPV on this tumor. There is a growing body of evidence suggesting that oropharyngeal HPV-HNSCC is a distinctive clinicopathological and molecular entity [33].

The two polymorphisms of MDM2 (309SNP TT and GT/GG) were analyzed on the basis of the seropositivity o negativity. Chen et al. (2010) conducted a case-control study finding an association between TT genotypes and HPV16 L1 seronegativity for OSCC risk and GT/GG and HPV16 L1 seropositivity (OR = 1.25, 95% CI = 1.06–2.19) and 2.81 (95% CI = 1.67–4.74). The authors reported that OR was 5.57 (95% CI = 2.93–10.6) for those with both the TT genotype and HPV16 L1 seropositivity. Similar results were observed for the MDM2 SNP2164 (AA and AG/GG) polymorphism. Moreover, there was a borderline significant interaction between the individual or combined MDM2 genotypes of the two polymorphisms and HPV16 L1 seropositivity on risk of OSCC (*P*(int⁡) = 0.060 for MDM2 SNP309, *P*(int⁡) = 0.009 for MDM2 SNP2164, and *P*(int⁡) = 0.005 for the combined MDM2 genotypes). Notably, the effects of MDM2 mutations were particularly pronounced in never smokers and never drinkers, and for oropharyngeal as opposed to oral cavity cancer. These results underline the central role of MDM2 in head and neck cancer development and suggest a correlation between MDM2 and HPV16 status. In the same study, the risk of OSCC has been associated with HPV16 L1 seropositivity, and it is modified by MDM2 promoter polymorphisms [34].

## 5. EGFR and p53/MDM2 Pathway

The epidermal growth factor receptor (EGFR) and its ligands are fundamentally important for cell division, migration, adhesion, invasion, and angiogenesis. EGFR overexpression has been reported in >80% of HNSCC tumors and is found to be associated with a more aggressive phenotype, poor prognosis, and resistance to chemotherapeutic agents [35].

To our knowledge, no direct correlations have been reported among p53/MDM2 polymorphism and EGFR. Although they are considered in few studies as a panel of biological markers both predictive for chemotherapy response and HNSCC risk.

Prado et al. (2010) reported a correlation between mRNA expression levels of COX-2, EGFR and p53 tested in oral leukoplakias. There were not correlations with sex, age and localization of the leukoplakia. However, by means of nonparametric statistics (Spearman's rank correlation coefficient), a positive linear correlation was found between EGFR and p53 mRNA expression levels (Spearman rho = 0.6, *n* = 24, *P* = 0.003) [36, 37].

## 6. Future Perspectives: p53 Target Therapy

Alterations in p53 gene have been associated to an aggressive phenotype; therefore, many preclinical and clinical trials investigated the way to restore wild type p53. Two approaches have reached more convincing results: p53 targeting small molecules and gene therapies [39].

During the past several years, a number of small-molecule, p53-MDM2-binding inhibitors have been developed in order to restore p53 activity. Nutlin-3 is a potent and selective small-molecule MDM2 antagonist that has shown considerable promise in preclinical studies [40]. Another approach has been studied by develop an adenovirus (ONYX-015) specifically engineered to selectively replicate and lyse p53-deficient cells and spare normal cells [41, 42].

P53 reactivating (p53RA) small molecules have been tested in head and neck squamous cell lines; they induced apoptosis with a dose-dependent increase in p53 protein expressions, resulting in upregulation of p21^waf1^ and activation of the intrinsic apoptotic pathway [39]. Roh et al. (2011) tested four molecules (PRIMA1, CP31398, RITA, and nutlin 3), showing that each p53RA effectively restored p53 function. Additional data on cell lines suggested a synergistic anti tumor effect [41].

Selective intratumoral replication and tumor-selective tissue destruction have been documented in phase I and II clinical trials of intratumoral injections of ONYX-015 with or without chemotherapy in patients with recurrent or refractory HNSCC [42].

Another approach of targeting p53 mutations involves gene replacement using a replication-defective adenoviral vector containing the wild-type p53 gene (Ad-p53 or RPR-INGN-201). In preclinical studies, Ad-p53 vector system has been shown to induce apoptosis in neoplastic cells regardless of their p53 status and to reduce tumor growth in mouse xenografts. In a phase I study, good tolerability was reported and a phase II study combining the virus with cytotoxic chemotherapy achieved some tumor response. Further studies on p53 target therapy are on going [43].

## 7. Discussion

The prognostic and predictive role of p53 has been confirmed, while some concerns on the reliability of MDM2 mutation depend on the presence of a few data on it.

In the general population, an increased risk or an earlier age of onset of HNSCC has been reported for patients with p53 R72P, while the association between age of onset and prognosis in patients with MDM2 SNP309 need, further confirmations. However, it appears that MDM2 and p53 together could be used as predictive markers of response but neither p53 alone nor MDM2 correlates with patients overall survival.

In fact, several studies reported that only the combination of p53 positive with concurrent low MDM2 resulted in a statistically significant association with reduced survival [21, 30, 31, 38].

Therefore, a new biomarker, the combination of p53 positive and MDM2 low level, should be considered for its prognostic role. Further studies on the role of p53/MDM2 are warranted especially in HPV positive HNSCC. In conclusion, the field of epidemiology has traditionally evaluated whether lifestyle factors are associated with HNSCC risk. However, with an increasing understanding of the molecular processes that underlie carcinogenesis, the field of molecular epidemiology has emerged. The ultimate goal of this progress is to personalize the approach for each patient. There is a need to identify drug-specific predictive biomarkers in order to better tailor chemotherapy regimens to individual patients with head and neck cancer. In cell and animal models, p53 is the critical factor for the outcome of genotoxic stress, such as the one triggered by many cancer therapies. The research and study of biological markers with phenotypic and functional perspectives and clinical application is designed to acquire basic information in order to better know and understand the natural history of preclinical and clinical tumor, without neglecting the practical aspects related to the identification of prognostic and predictive indicators of different types of local or systemic treatment. P53 and MDM2 are both important players in the DNA damage repair. Detection of MDM2 protein expression by immunohistochemical may be an important diagnostic tool in the future. Some mutations of both p53 and MDM2 correlate with worst prognosis. The application of molecular predictors of clinical outcome would be extremely useful to allow rational selection of patients who are more likely to benefit from treatment and to spare unnecessary toxicity to those with a poor chance of response. A model which correlate several markers (EGFR status, HPV, p53 mutations, and MDM2 status) with old prognostic known factors may allow a more targeted therapy.

## Figures and Tables

**Table 1:** Some of the most impressive studies on p53-MDM2 correlations.

Author	Pts	Biological predictors/prognosticators	Correlations	p
Michalides et al. [28]	198	MDM2, MTBP, p53, and HNSCC OS*	Low MDM2 versus high MDM2	0.248
			P53+ AND low MDM2 versus other status	0.035

Poeta et al. [29]	53	P53 and RR after P-based NACT	4/15 CR versus 20/38 NR	0.12

Nakashima et al. [24]	76	P53, MDM2, Rb, and dysplasia	HNSCC	P53 66% MDM2 46%
			Hyperplastic lesions	P53 55% MDM2 31%
			Dysplastic lesion	P53 64% MDM2 44%

Hamid et al. [27]	62	MDM2, p53, HNSCC OS, and stage	P53+ AND low MDM2	0.07
			P53+ AND IHC staining	0.018
			MDM2 and T stage	0.10
			MDM2 and T4 versus T1	0.015
			Low MDM2 and 3 y OS	0.034

Chen et al. [34]	660**	MDM2 polymorphism and HPV 16 seropositivity	MDM2-rs2279744 TT versus GT	OR = 0.64
			MDM2-rs2279744 TT versus GG	OR = 0.60
			MDM2-rs2279744 TT versus GT/GG	OR = 0.62
			MDM2-rs2279744 TT versus AG	OR = 2.20
			MDM2-rs2279744 TT versus AG/GG	OR = 2.05

Gasco and Crook [17]	2073***	MDM2, p53, and HNSCC onset	MDM2 SNP309	0.638
			MDM2 A2164G	0.580
			P53 codon 72	0.193

Agarwal et al. [38]	128****	MDM2, p53, and prognosis	MDM2 mut/p53+ AND stage III/IV	0.0009
			MDM2 mut/p53+ AND N+	0.0325

Sullivan et al. [20]	73	p53 and OS	P53 72R versus p53 72P	0.007
			wt p53 (72R or 72P) versus no wt P53	0.0001
			P53 72R versus p53 72P + 72R	0.02

Bergamaschi et al. [5]	70	p53, p73, OS, and response	CR: p53 wt versus no wt P53	0.0001
			PFS: p53 wt versus no wt P53	0.0007
			PFS: p53 72R versus 72P	0.008
			OS: p53 72R versus 72P	0.0044
			PFS: p73−/p53+ versus p73+/p53+	0.05

Hamid et al. [27]	420	p53 and OS	p53+ versus p53−	0.074
			p53+ disruptive versus p53+ nondisruptive	0.001
				RR = 1.17

*= OS after surgery; **= 335 controls; ***= 1090 control population; ****= 33 premalignant lesion and 30 control normal oral tissues.

OS = overall survival; NACT = neoadjuvant chemotherapy; OR = odd ratio; RR = response rate; CR = complete response.

IHC = immuhistochemical; NR = nonresponse;

3y = 3 year; p53+ = p53 mutated; p53 codon 72 = mutation in codon 72.

72R = arginine at codon 72 of p53.

72P = proline at codon 72 of p53.

p73−/p53+ = non-p73-inactivating p53 mutations. p73+/p53+ = p73-inactivative and p53 mutations.
